# Grass species with potential for rangelands restoration in northern Mexico: an assessment with environmental niche modeling

**DOI:** 10.1038/s41598-024-56918-1

**Published:** 2024-03-15

**Authors:** Alan Álvarez-Holguín, Carlos Raúl Morales-Nieto, Raúl Corrales-Lerma, Jesús Manuel Ochoa-Rivero, Omar Castor Ponce-García, Jesús Alejandro Prieto-Amparán, José Humberto Vega-Mares, Federico Villarreal-Guerrero

**Affiliations:** 1 Facultad de Zootecnia y Ecología, Universidad Autónoma de Chihuahua, Periférico Francisco R. Almada Km 1, 31453 Chihuahua, Chih. Mexico; 2 Campo Experimental La Campana, Instituto Nacional de Investigaciones Forestales, Agrícolas y Pecuarias (INIFAP), Carretera Chihuahua-Ojinaga Km. 33.3, 32190 Aldama, Chih. Mexico

**Keywords:** Ecology, Ecological modelling, Ecosystem ecology, Grassland ecology, Restoration ecology

## Abstract

Environmental niche modeling (ENM) has emerged as a promising tool for identifying grass species with potential for rangeland restoration. This approach can detect suitable areas and environments where these species can be planted. In this study, we employed ENM to estimate the potential distribution range of 50 grass species of the grasslands and shrublands of northern Mexico. The outcome of the ENM served to identify grass species with potential for restoration in Mexico, especially those not commonly used for that purpose in the past. Results suggested the possibility of selecting seven grass species with the potential for revegetating degraded grasslands, nine for shrublands, and six for alkaline soils. This research provides insights into the environmental adaptations of different grass species distributed in the rangelands of northern Mexico. Ecologists, conservation planners, researchers, and range managers could use these outcomes and the maps of the potential distribution ranges as supportive information to conduct effective restoration efforts. In turn, this can assist in increasing the probability of success of future rangelands restoration programs, which are often costly in terms of financial investments and labor.

## Introduction

Rangelands encompass a variety of ecosystems, including grasslands, shrublands, and some types of forests and jungles. Rangelands can be defined as large areas dominated by grasses, shrubs and non-forest vegetation, covering about 50% of the earth’s land surface^1^. Thus, they play a crucial role in supporting ecosystem services, such as carbon sequestration, soil erosion control, livestock production, among others^2^. Although the extent of rangelands degradation over the world is hard to assess, concern exists that significant portions of the rangeland ecosystems are degraded^3^. To reverse such a degradation, different programs have been undertaken including revegetation, which has been intensified globally over the last decade^4^.

Revegetation is a crucial tool for restoring degraded rangelands^5^. It involves planting or seeding suitable plant species to increase plant cover and improve the ecosystem’s functions. Grasses play a vital role in the revegetation of rangelands; however, their establishment can be challenging^4,6,7^. One of the main challenges for the establishment of grasses during rangelands restoration is the harsh environmental conditions of degraded areas. In northern Mexico, rangelands are often characterized by low rainfall, high temperatures, and poor soil conditions. These conditions can make it difficult for newly planted grasses to establish and survive. For this reason, rangeland restoration requires the identification of suitable plant species, which can be successfully established under harsh environmental conditions.

Environmental niche modeling (ENM) has emerged as a promising tool for identifying potential plant species for rangeland restoration based on the species’ environmental adaptability^8–10^. This approach uses ecological data and statistical modeling to predict the environmental suitability of plant species^11,12^. In turn, predicting environmental niche of grass species can serve for biodiversity conservation and rehabilitation of rangeland ecosystems^13^. This information is also useful for restoration efforts since it identifies areas with suitable environments where the species can be planted^14^. In this sense, this approach can be used to detect species with potential for rangelands restoration, which have not been commonly used for that purpose in the past, expanding the range of restoration options. Therefore, the objectives of the present study were (1) to model the environmental suitability of 50 grass species distributed in the grasslands and shrublands of northern Mexico and to determine their potential distribution range; (2) to evaluate the clustering pattern of the grass species based on the environmental conditions of their distribution range; and (3) to identify the species, which could be used to restore degraded rangelands of northern Mexico.

## Materials and methods

### Focal species and occurrence data

The identification process started by reviewing the literature about the grass species, which are native to northern Mexico^15–17^. Based on that, we pre-selected 168 perennial grass species distributed in the grasslands and shrublands of northern Mexico. For these species, we explored the occurrences data in the Global Biodiversity Information Facility (GBIF; https://www.gbif.org/es/). From the GBIF data, we selected only the species having 80 occurrence records or more, which resulted in a subset of 50 native perennial species (Table 1). Occurrence records of these species were then downloaded from both, the GBIF and the ‘Comisión Nacional para el Conocimiento y Uso de la Biodiversidad’ (CONABIO; https://enciclovida.mx/) databases, and then used in further analyses. In addition, we used occurrence data recorded in the field by our research team (Supplementary Table S1). When more than one occurrence was registered within an area of 1 km^2^, only one was recorded and the rest were eliminated to avoid redundance. That was performed in the ‘spThin’ package of the R software ver. 4.1.2. The invasive exotic species *Pennisetum ciliare* (buffel grass) was included as a control species since it is the species utilized for rangelands revegetation in the arid and semiarid regions of central and northern Mexico^18^.

**Table 1:** Species and the number of occurrences used for the environmental niche modeling.

Species	Acronym	Common name	No. of occurrences	Species	Acronym	Common name	No. of occurrences
*Andropogon gerardii*	Ange	Big bluestem	129	*Erioneuron avenaceum*	Erav	Hortleaf woollygrass	162
*Aristida divaricata*	Ardi	Poverty threeawn	479	*Heteropogon contortus*	Heco	Tanglehead	231
*Aristida pansa*	Arpa	Wooton’s threeawn	200	*Hilaria mutica*	Himu	Tobosagrass	206
*Aristida purpurea*	Arpu	Purple threeawn	103	*Hopia obtusa*	Paob	Vine mesquite	369
*Aristida ternipes*	Arte	Spidergrass	217	*Leptochloa crinita*	Trcr	False Rhodes grass	74
*Bothriochloa barbinodis*	Boba	Cane bluestem	214	*Microchloa kunthii*	Miku	Kunth's smallgrass	111
*Bothriochloa laguroides*	Bola	Silver beardgrass	319	*Muhlenbergia arenicola*	Muar	Sand muhly	176
*Bouteloua chondrosioides*	Boch	Sprucetop grama	117	*Muhlenbergia glauca*	Mugl	Desert muhly	170
*Bouteloua curtipendula*	Bocu	Sideoats grama	475	*Muhlenbergia phleoides*	Muph	Common wolfstail	186
*Bouteloua dactyloides*	Boda	Buffalograss	121	*Muhlenbergia porteri*	Mupo	Bush muhly	176
*Bouteloua eriopoda*	Boer	Black grama	130	*Panicum hallii*	Paha	Hall's panicgrass	84
*Bouteloua gracilis*	Bogr	Blue grama	298	*Panicum hirticaule*	Pahi	Mexican panicgrass	148
*Bouteloua hirsuta*	Bohi	Hairy grama	234	*Pappophorum bicolor*	Pabi	Pink Pappusgrass	150
*Bouteloua radicosa*	Bora	Purple grama	89	*Paspalum distichum*	Padi	knotgrass	110
*Bouteloua ramosa*	Borm	Chino grama	174	*Pennisetum ciliare*	Peci	Buffelgrass	1213
*Bromus anomalus*	Bran	Nodding brome	154	*Schizachyrium scoparium*	Scsc	Little bluestem	174
*Chloris submutica*	Chsu	Mexican Windmill	109	*Scleropogon brevifolius*	Acbr	Burrograss	230
*Dasyochloa pulchella*	Dasy	Low woollygrass	120	*Setaria leucopila*	Sele	Streambed bristlegrass	93
*Digitaria californica*	Dica	Arizona cottontop	120	*Setaria macrostachya*	Sema	large-spike bristlegrass	91
*Digitaria cognata*	Dico	Fall witchgrass	83	*Setaria parviflora*	Sepa	Marsh bristlegrass	328
*Disakisperma dubium*	Ledu	Green sprangletop	159	*Sorghastrum nutans*	Sonu	Indiangrass	194
*Distichlis spicata*	Disp	Saltgrass	223	*Sporobolus airoides*	Spar	Alkali sacaton	80
*Elionurus barbiculmis*	Elba	Woolyspike balsamscale	183	*Sporobolus flexuosus*	Spfl	Mesa dropseed	65
*Enneapogon desvauxii*	Ende	Nineawn pappusgrass	105	*Trachypogon spicatus*	Trsp	Spiked crinkleawn	857
*Eragrostis intermedia*	Erin	Plains lovegrass	176	*Zuloagaea bulbosa*	Pabu	Bulb panicgrass	167
*Andropogon gerardii*	Ange	Big bluestem	129	*Erioneuron avenaceum*	Erav	Hortleaf woollygrass	162

### Environmental variables

Initially, the 19 bioclimatic variables from the Worldclim database (https://www.worldclim.org) were downloaded^19^. The variables of wind speed, solar radiation, and elevation were also obtained from the Worldclim 2.1 database ^20^. In addition, evapotranspiration and the aridity index were downloaded from the Consortium for Spatial Information (https://cgiarcsi.community)^21^. All these variables were obtained for the period 1970–2000 (historical climate data), at a resolution of 2.5 arc-min.

Besides climate variables, soil variables play a key role in the distribution of plant species^22,23^. Thus, the analysis also included the variables of texture (sand, clay and silt), together with calcium, carbon, potassium, magnesium, and sodium contents, as well as electrical conductivity, cation exchange capacity, and pH. These data were retrieved from 16,820 soil analyses performed along the country by the ‘Instituto Nacional de Estadística, Geografía e Informática’ (INEGI, 2010). Soil data were interpolated through the Kriging’s method, using ArcMap ver. 10.3 (ESRI, CA). The outcomes were obtained at a resolution of 2.5 arc-min. Interpolations were validated by dividing the data into 90/10% train test. The measured and estimated values were then compared by using the t-Student test (α = 0.05) in the R software ver. 4.1.2. No differences were found (*p* > 0.05) in all the soil variables interpolated. Therefore, they were included in the analyses.

### Data analysis

Once all the variables were obtained, they were subjected to a Pearson’s correlation test to identify and exclude highly correlated variables. As a result, nine variables were discarded because they showed a degree of collinearity (Pearson’s correlation coefficient) higher than 0.8, leaving 26 variables. The bioclimatic variables included in the analyses were mean diurnal range (Bio2), isothermality (Bio3), temperature annual range (Bio7), mean temperature of the warmest quarter (Bio10), mean temperature of the coldest quarter (Bio11), annual precipitation (Bio12), precipitation seasonality (Bio15), precipitation of the driest quarter (Bio17), elevation, evapotranspiration, aridity index, solar radiation, and wind speed. The soil variables sand, clay, silt, calcium, carbon, potassium, magnesium, and sodium contents, together with electrical conductivity, cation exchange capacity, and pH were also included in the analyses.

Cluster and principal component analyses were carried out to evaluate if the grass species presented a clustering pattern based on the environmental conditions of their distribution range. Cluster analysis was conducted following the Ward’s method and the optimal number of groups was established based on the k-means approach. ArcMap ver. 10.3 (ESRI, CA) was utilized to sample the climate and soil data for each presence record of all the grass species. Then, mean values of the climate and soil variables were calculated for the records of each species and used to perform the cluster and principal component analyses. The analyses were performed using the R software ver. 4.1.2.

Environmental niche modeling was applied to identify grass species with potential for rangelands restoration in northern Mexico. This analysis was performed through the maximum entropy model approach using MaxEnt ver. 3.4.4^11,12^. The presence data and the climate and soil variables were used as input for the modeling. The MaxEnt program offers five features: Linear (L), quadratic (Q), product (P), threshold (T) and hinge (H). These features were used to develop 5 MaxEnt models per species. This was performed by using the ENMeval package in the R software^24^, setting the regularization multiplier as default. The best model per species was selected using two criteria: first, the models were separated based on partial ROC tests^25^, discarding non-significant models (*p* > 0.1). Second, the remaining models were sorted by the Akaike Information Criterion corrected for small sample sizes (AICc), choosing the one with the lowest value as the final model^26^. Models were constructed by using 10 repeated runs, with a maximum number of interactions of 5,000 and 10,000 background pseudo-absence points. The models were built with 75% of the sampling points; the remaining 25% were randomly selected as test data. The bootstrap method was set as the replicated run type. The model performance was evaluated based on the Area Under the Receiver Operating Curve (AUC)^27^. MaxEnt outputs were transformed into geographic maps in ArcMap ver. 10.3 (ESRI, CA). The maps show the environmental niche of the grass species based on an index of suitability between 0 and 1, where 0 indicates unsuitable environmental conditions and 1 indicates suitable conditions.

## Results

### Cluster association

**Figure 1:**
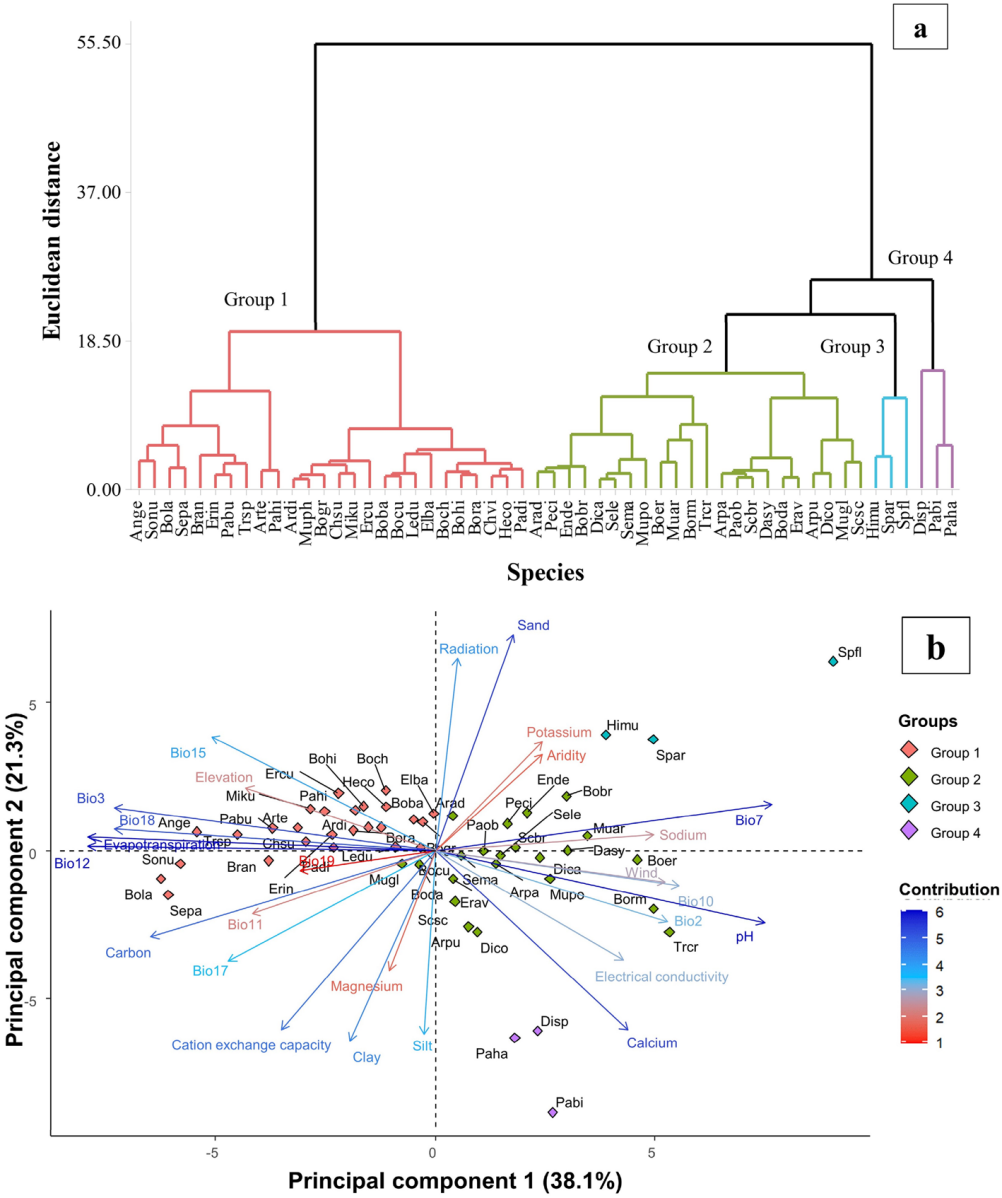
Clustering patterns of 50 grass species developed with the means of 26 environmental variables, obtained from their distribution range (**a**). Biplot of the first two principal components showing the distribution of the environmental variability for the distribution of the 50 grass species (**b**). Arrow lines illustrate the loadings of each variable (WorldClim Bio1, Bio3, Bio7, Bio10, Bio11-12, Bio15, Bio17-19, elevation, evapotranspiration, radiation, aridity index, wind speed) and soil variables. Colors (red to blue) indicate the percentage of contribution of the variables to the principal components. *Andropogon gerardii* = Ange, *Aristida divaricata* = Ardi, *Aristida pansa* = Arpa, *Aristida purpurea* = Arpu, *Aristida ternipes* = Arte, *Bothriochloa barbinodis* = Boba, *Bothriochloa laguroides* = Bola, *Bouteloua chondrosioides* = Boch, *Bouteloua curtipendula* = Bocu, *Bouteloua dactyloides* = Boda, *Bouteloua eriopoda* = Boer, *Bouteloua gracilis* = Bogr, *Bouteloua hirsuta* = Bohi, *Bouteloua radicosa* = Bora, *Bouteloua ramosa* = Borm, *Bromus anomalus* = Bran, *Pennisetum ciliare* = Peci, *Chloris submutica* = Chsu, *Dasyochloa pulchella* = Dasy, *Digitaria californica* = Dica, *Digitaria cognata* = Dico, *Elionurus barbiculmis* = Elba, *Enneapogon desvauxii* = Ende, *Eragrostis curvula* = Ercu, *Eragrostis intermedia* = Erin, *Erioneuron avenaceum* = Erav, *Heteropogon contortus* = Heco, *Hilaria mutica* = Himu, *Disakisperma dubium* = Ledu, *Muhlenbergia phleoides* = Muph, *Microchloa kunthii* = Miku, *Muhlenbergia arenicola* = Muar, *Muhlenbergia glauca* = Mugl, *Muhlenbergia porteri* = Mupo, *Zuloagaea bulbosa* = Pabu, *Panicum hirticaule* = Pahi, *Hopia obtusa* = Paob, *Paspalum distichum* = Padi, *Schizachyrium scoparium* = Scsc, *Scleropogon brevifolius* = Acbr, *Setaria leucopila* = Sele, *Setaria macrostachya* = Sema, Setaria parviflora = Sepa, *Sorghastrum nutans* = Sonu, *Sporobolus airoides* = Spar, *Sporobolus flexuosus* = Spfl, *Trachypogon spicatus* = Trsp, *Leptochloa crinita* = Trcr, *Distichlis spicata* = Disp, *Panicum hallii* = Paha, *Pappophorum bicolor* = Pabi.

Cluster analysis separated the grass species into four groups (Fig. 1). Species distributed in areas with the highest isothermality (Bio3), annual precipitation (Bio12), precipitation seasonality (Bio15), precipitation of the driest quarter (Bio17), precipitation of the warmest quarter (Bio18), evapotranspiration and organic carbon content were clustered in Group 1. Species distributed in areas with the highest mean diurnal range (Bio2), mean temperature of the warmest quarter (Bio10), wind speed, pH, sodium, calcium, and electrical conductivity were clustered in Groups 2, 3, and 4. Group 3 was shaped by species distributed in areas with the highest temperature annual range (Bio7), radiation, and sand content while Group 4 clustered species adapted to the highest cation exchange capacity and the highest magnesium, calcium, and clay contents.

### Environmental niche modeling

Distribution models showed high accuracy with values of AUC greater than 0.85. Most of the models (90%) showed an accuracy greater than 0.9, with an average AUC of 0.93 (Table 2). Therefore, the accuracy of the constructed models was high and could be used to predict the habitat suitability range of the species analyzed. The species with the highest habitat suitability range (greater than 75%) were *Bouteloua curtipendula* (118,830 km^2^), followed by *Trachypogon secundus* (103,243 km^2^), *Leptochloa crinita* (89,332 km^2^), *Bouteloua hirsuta* (87,995 km^2^), *Aristida divaricata* (75,699 km^2^), *Hopia obtusa* (69,740 km^2^), *Bouteloua gracilis* (68,496km^2^), *Aristida ternipes* (64,875 km^2^), and *Setaria parviflora* (57,756 km^2^). Elevation was the variable with the highest contribution for 25 of the 50 grass species evaluated. Aridity was the variable with the highest contribution for *Hilaria mutica, Dasyochloa pulchella, Sorghastrum nutans,* and *Trachypogon secundus*. Likewise, temperature annual range was the variable with the highest contribution for *Digitaria cognata*, *Digitaria californica*, *Muhlenbergia porteri*, *Setaria parviflora*, *Bouteloua eriopoda*, and *Sporobolus flexuosus*. Soil variables such as calcium were the most important for *Aristida purpurea* and *Panicum hallii,* while pH was the most important factor for the habitat suitability of *Setaria leucopila* (Table 3).

**Table 2:** Surface with a probability of occurrence greater than 75% for the distribution of 50 grass species, based on MaxEnt environmental niche models.

Species	AUC	Area > 0.75 (km^2^)	Species	AUC	Area > 0.75 (km^2^)
*Bouteloua curtipendula*	0.86 ± 0.01	118,830	*Panicum hallii*	0.94 ± 0.01	36,323
*Trachypogon secundus*	0.86 ± 0.01	103,243	*Muhlenbergia phleoides*	0.94 ± 0.01	34,409
*Leptochloa crinita*	0.96 ± 0.01	89,332	*Digitaria californica*	0.93 ± 0.01	34,381
*Bouteloua hirsuta*	0.89 ± 0.01	87,995	*Zuloagaea bulbosa*	0.93 ± 0.01	34,235
*Aristida divaricata*	0.90 ± 0.01	75,699	*Schizachyrium scoparium*	0.94 ± 0.01	33,974
*Hopia obtusa*	0.95 ± 0.01	69,740	*Setaria leucopila*	0.92 ± 0.01	33,805
*Bouteloua gracilis*	0.90 ± 0.01	68,496	*Andropogon gerardii*	0.95 ± 0.01	33,190
*Aristida ternipes*	0.90 ± 0.01	64,875	*Scleropogon brevifolius*	0.94 ± 0.01	33,165
*Pennisetum ciliare*	0.90 ± 0.01	61,272	*Enneapogon desvauxii*	0.93 ± 0.01	31,845
*Setaria parviflora*	0.88 ± 0.01	57,756	*Bouteloua ramosa*	0.96 ± 0.01	29,686
*Bothriochloa barbinodis*	0.90 ± 0.01	51,507	*Panicum hirticaule*	0.91 ± 0.01	28,899
*Heteropogon contortus*	0.87 ± 0.01	51,322	*Bromus anomalus*	0.95 ± 0.01	28,290
*Aristida pansa*	0.93 ± 0.01	49,195	*Bouteloua eriopoda*	0.95 ± 0.01	27,785
*Eragrostis intermedia*	0.93 ± 0.01	48,851	*Muhlenbergia porteri*	0.94 ± 0.01	26,800
*Bothriochloa laguroides*	0.92 ± 0.01	45,851	*Pappophorum bicolor*	0.96 ± 0.01	25,945
*Bouteloua radicosa*	0.93 ± 0.01	45,610	*Microchloa kunthii*	0.95 ± 0.01	25,697
*Setaria macrostachya*	0.92 ± 0.01	43,711	*Paspalum distichum*	0.94 ± 0.01	25,650
*Dasyochloa pulchella*	0.94 ± 0.01	42,934	*Muhlenbergia glauca*	0.95 ± 0.01	23,241
*Bouteloua chondrosioides*	0.95 ± 0.01	42,890	*Muhlenbergia arenicola*	0.96 ± 0.01	22,323
*Erioneuron avenaceum*	0.95 ± 0.01	41,467	*Sporobolus airoides*	0.94 ± 0.01	22,228
*Elionurus barbiculmis*	0.94 ± 0.01	39,353	*Distichlis spicata*	0.94 ± 0.01	21,584
*Bouteloua dactyloides*	0.96 ± 0.01	39,173	*Chloris submutica*	0.96 ± 0.01	20,295
*Sorghastrum nutans*	0.94 ± 0.01	39,157	*Digitaria cognata*	0.96 ± 0.01	19,521
*Disakisperma dubium*	0.92 ± 0.01	38,230	*Sporobolus flexuosus*	0.99 ± 0.01	7553
*Aristida purpurea*	0.95 ± 0.01	36,660	*Hilaria mutica*	0.98 ± 0.01	4584

*AUC=* Area under the curve.

**Table 3:** Variables with the highest contribution for the environmental niche models of 50 grass species.

Species	Variable with the highest contribution	Species	Variable with the highest contribution
*Andropogon gerardii*	Elevation (37%)	*Erioneuron avenaceum*	Elevation (38.6%)
*Aristida divaricata*	Elevation (50.6%)	*Heteropogon contortus*	Precipitation seasonality (16.1%)
*Aristida pansa*	Elevation (35.3%)	*Hilaria mutica*	Aridity (15.1%)
*Aristida purpurea*	Calcium (21.9%)	*Hopia obtusa*	Elevation (50.1%)
*Aristida ternipes*	Precipitation seasonality (23.5%)	*Leptochloa crinita*	Radiation (28.3%)
*Bothriochloa barbinodis*	Elevation (18.5%)	*Microchloa kunthii*	Elevation (31.1%)
*Bothriochloa laguroides*	Elevation (17.0%)	*Muhlenbergia arenicola*	Elevation (32.2%)
*Bouteloua chondrosioides*	Elevation (18.8%)	*Muhlenbergia glauca*	Elevation (41.7%)
*Bouteloua curtipendula*	Elevation (25.4%)	*Muhlenbergia phleoides*	Elevation (56.3%)
*Bouteloua dactyloides*	Elevation (23.2%)	*Muhlenbergia porteri*	Temperature annual range (21.3%)
*Bouteloua eriopoda*	Temperature annual range (25.9%)	*Panicum hallii*	Calcium (26.2%)
*Bouteloua gracilis*	Elevation (51.8%)	*Panicum hirticaule*	Precipitation seasonality (21.9%)
*Bouteloua hirsuta*	Elevation (32.6%)	*Pappophorum bicolor*	Radiation (37.0%)
*Bouteloua radicosa*	Elevation (13.8%)	*Paspalum distichum*	Elevation (16.2%)
*Bouteloua ramosa*	Radiation (32.7%)	*Pennisetum ciliare*	Precipitation seasonality (13.4%)
*Bromus anomalus*	Elevation (43.6%)	*Schizachyrium scoparium*	Precipitation of Driest Quarter (21.1%)
*Chloris submutica*	Elevation (47.9%)	*Scleropogon brevifolius*	Elevation (43.0%)
*Dasyochloa pulchella*	Aridity (23.3%)	*Setaria leucopila*	pH (16.1%)
*Digitaria californica*	Temperature annual range (19.3%)	*Setaria macrostachya*	Radiation (14.0%)
*Digitaria cognata*	Temperature annual range (11.4%)	*Setaria parviflora*	Temperature annual range (25.5%)
*Disakisperma dubium*	Elevation (29.6%)	*Sorghastrum nutans*	Aridity (25.3%)
*Distichlis spicata*	Precipitation of warmest quarter (19.0%)	*Sporobolus airoides*	Mean Temperature of Coldest Quarter (18.6%)
*Elionurus barbiculmis*	Elevation (31.6%)	*Sporobolus flexuosus*	Temperature annual range (42.6%)
*Enneapogon desvauxii*	Annual precipitation (23.8%)	*Trachypogon secundus*	Aridity (28.9%)
*Eragrostis intermedia*	Elevation (37.8%)	*Zuloagaea bulbosa*	Elevation (40.2%)

### Alternatives to revegetate degraded grasslands

**Figure 2:**
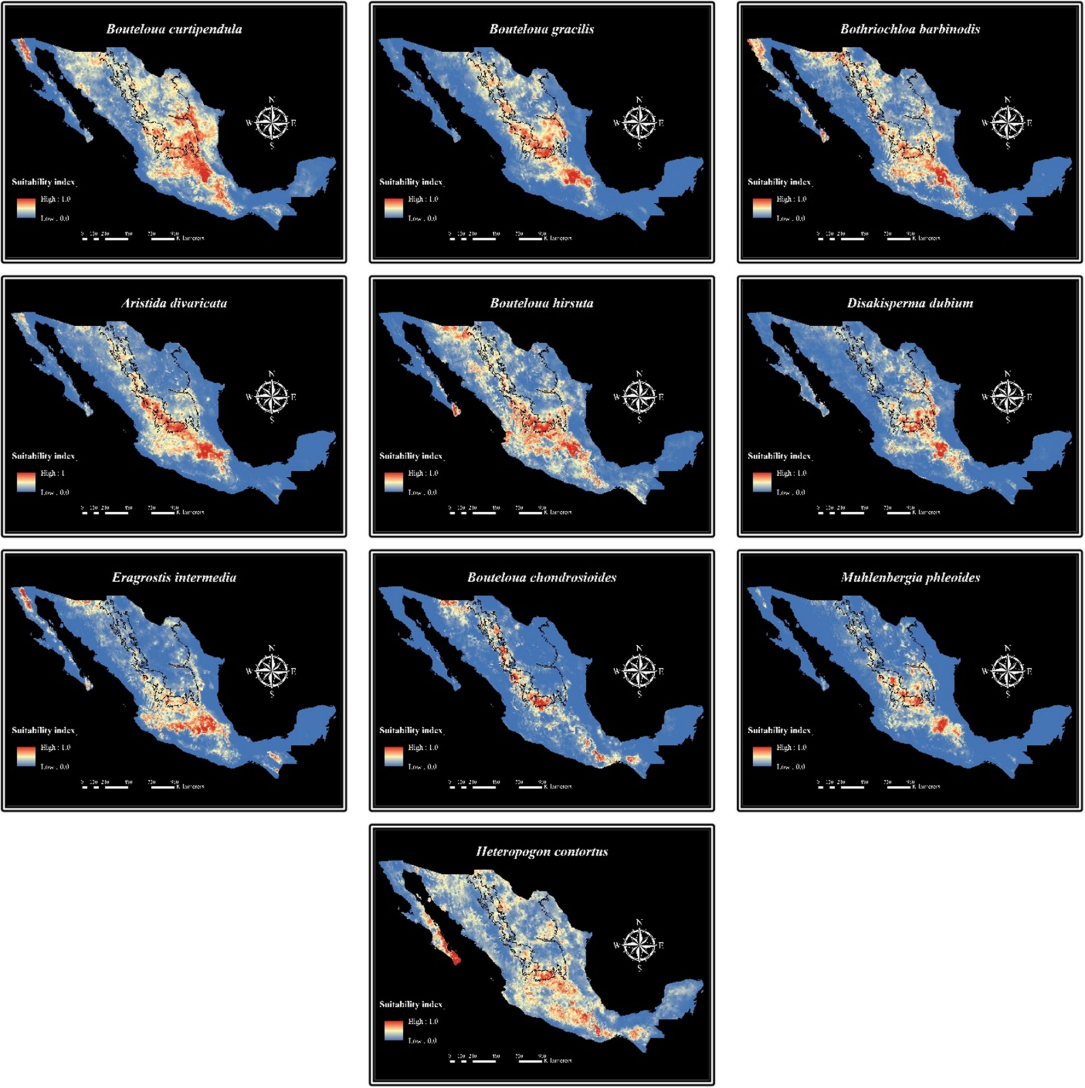
MaxEnt niche models for the potential distribution of 10 grass species, which could be used for rangelands restoration in the grasslands of northern Mexico. Relative environmental suitability for the species ranges from 0 (blue) to 1 (red). Black lines illustrate the grasslands (left) and shrublands (right) distribution ranges.

Species from Group 1 (Fig. 1) could be used to revegetate degraded grasslands since they are distributed in areas with the highest precipitation and organic carbon content. This agrees with the environmental niche models obtained in MaxEnt. The suitability index of the species included in Group 1 was higher in the grasslands than in the shrublands region. Figure 2 shows the habitat suitability range of 10 species belonging to Group 1. These species registered the highest surfaces with a probability of occurrence greater than 75%.

### Alternatives to revegetate degraded shrublands

**Figure 3:**
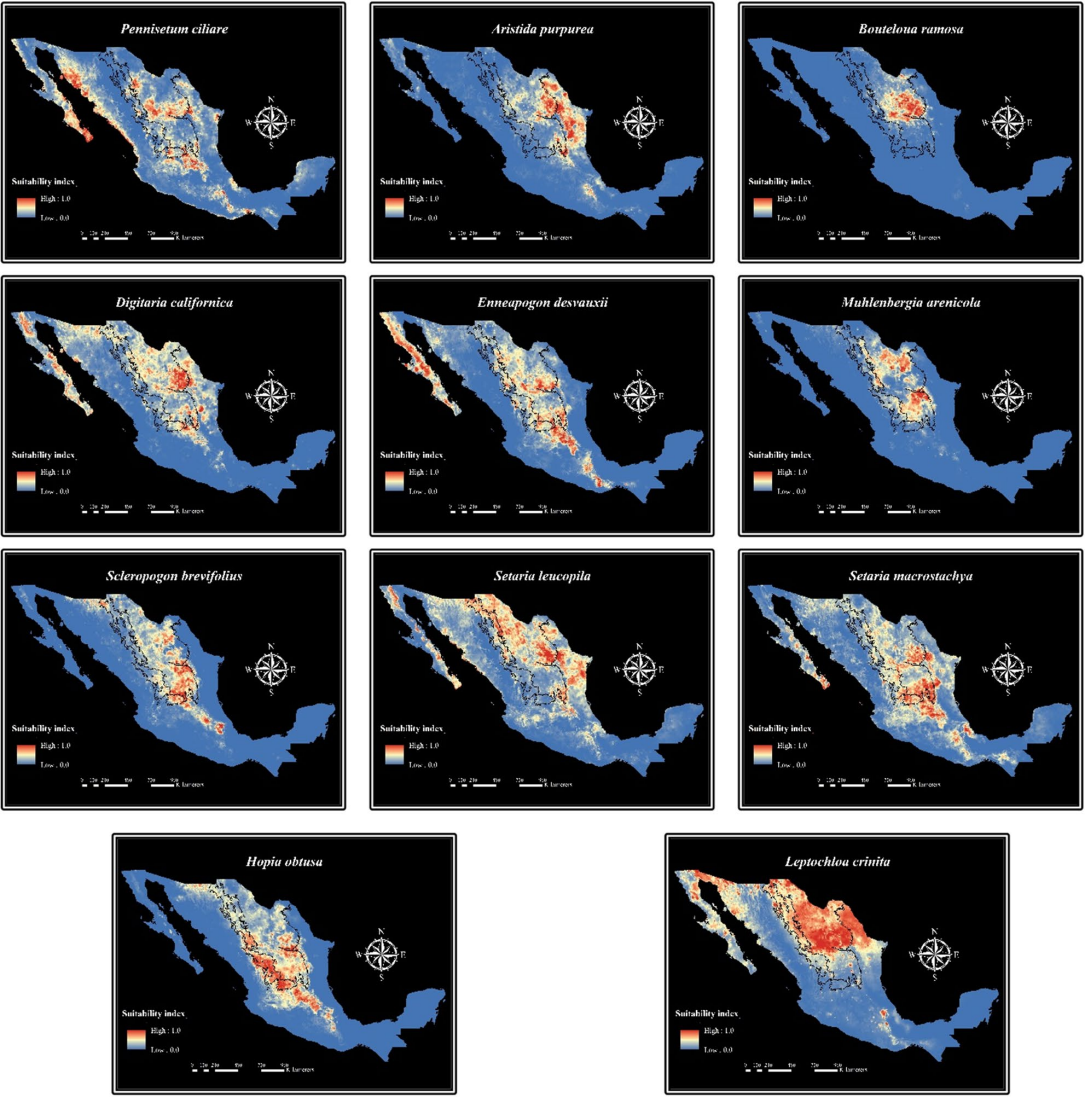
MaxEnt niche models for the potential distribution of 10 grass species that can be used for rangelands restoration in shrublands of Northern Mexico. Relative environmental suitability for the species ranges from 0 (blue) to 1 (red). Black lines illustrate the grasslands (left) and shrublands (right) distribution ranges.

Group 2 of Fig. 1 was shaped by species adapted to low rainfall, high temperatures, and alkaline soils. This suggests the species from this group can be used to revegetate shrublands. That is in agreement with the results of the environmental niche models. Species included in Group 2 had higher suitability in the shrublands region than in the grasslands region (Fig. 3). *Pennisetum ciliare* (buffel grass) was included as a control species since it is an alien and invasive species. This species presented a habitat suitability range of 61,272 km^2^ (areas with a suitability greater than 75%). Two native species presented a broader habitat suitability range than *P. ciliare*; they were: *Leptochloa crinita* (89,332 km^2^) and *Hopia obtusa* (69,740 km^2^). In addition, *Aristida purpurea*, *Bouteloua ramosa*, *Digitaria californica*, *Enneapogon desvauxii*, *Muhlenbergia arenicola*, *Scleropogon brevifolius*, *Setaria leucopila,* and *Setaria macrostachya* showed a great habitat suitability range in the shrublands region. This suggests these species may be an alternative to *Pennisetum ciliare* and can be used to revegetate degraded shrublands in northern Mexico.

### Alternatives to revegetate under alkaline soil conditions

Species clustered in groups 3 and 4 (Fig. 1) are distributed in areas with the highest mean diurnal range (Bio2), mean temperature of the warmest quarter (Bio10), wind speed, pH, sodium, calcium, and electrical conductivity. Therefore, they could be used to restore arid lands with alkaline soils. On the one hand, Group 3 was shaped by *Hilaria mutica*, *Sporobolus airoides*, and *Sporobolus flexuosus*, which can be found in the areas with the highest sand content. Accordingly, the variables pH, calcium, sodium, and sand showed an important contribution to the ENMs of these species (Supplementary Table S2). These results suggest that species from Group 3 are adapted to sandy-alkaline soils and may serve to revegetate areas with this type of soil. On the other hand, Group 4 was clustered by *Distichlis spicata*, *Pappophorum bicolor*, and *Panicum hallii*. These species can be located in the areas with the highest cation exchange capacity, as well as the highest magnesium, calcium, slit, and clay contents, suggesting they could be used in clayey and silty alkaline soils.

**Figure 4:**
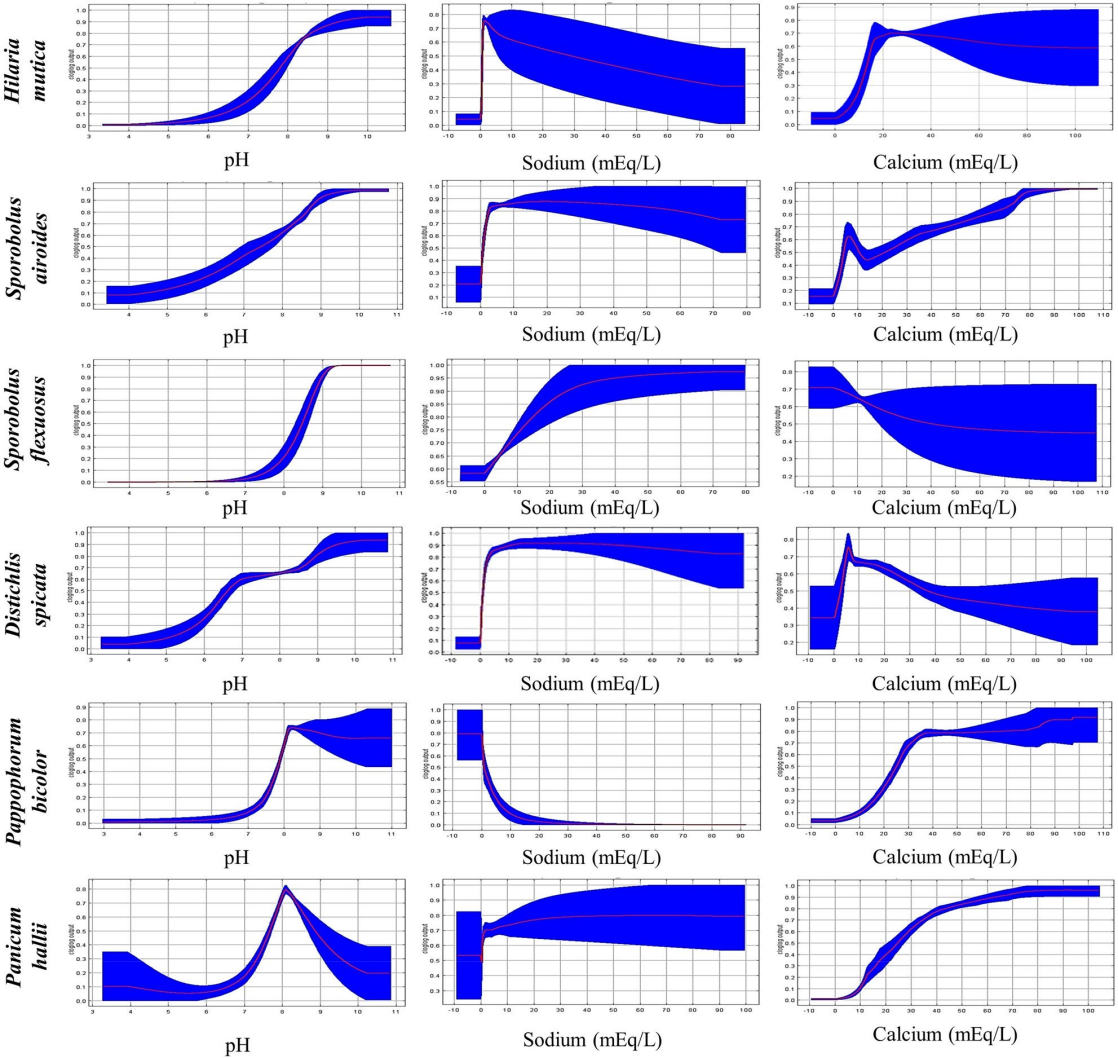
Response curves of pH, sodium, and calcium for the environmental niche models of six grass species.

**Figure 5:**
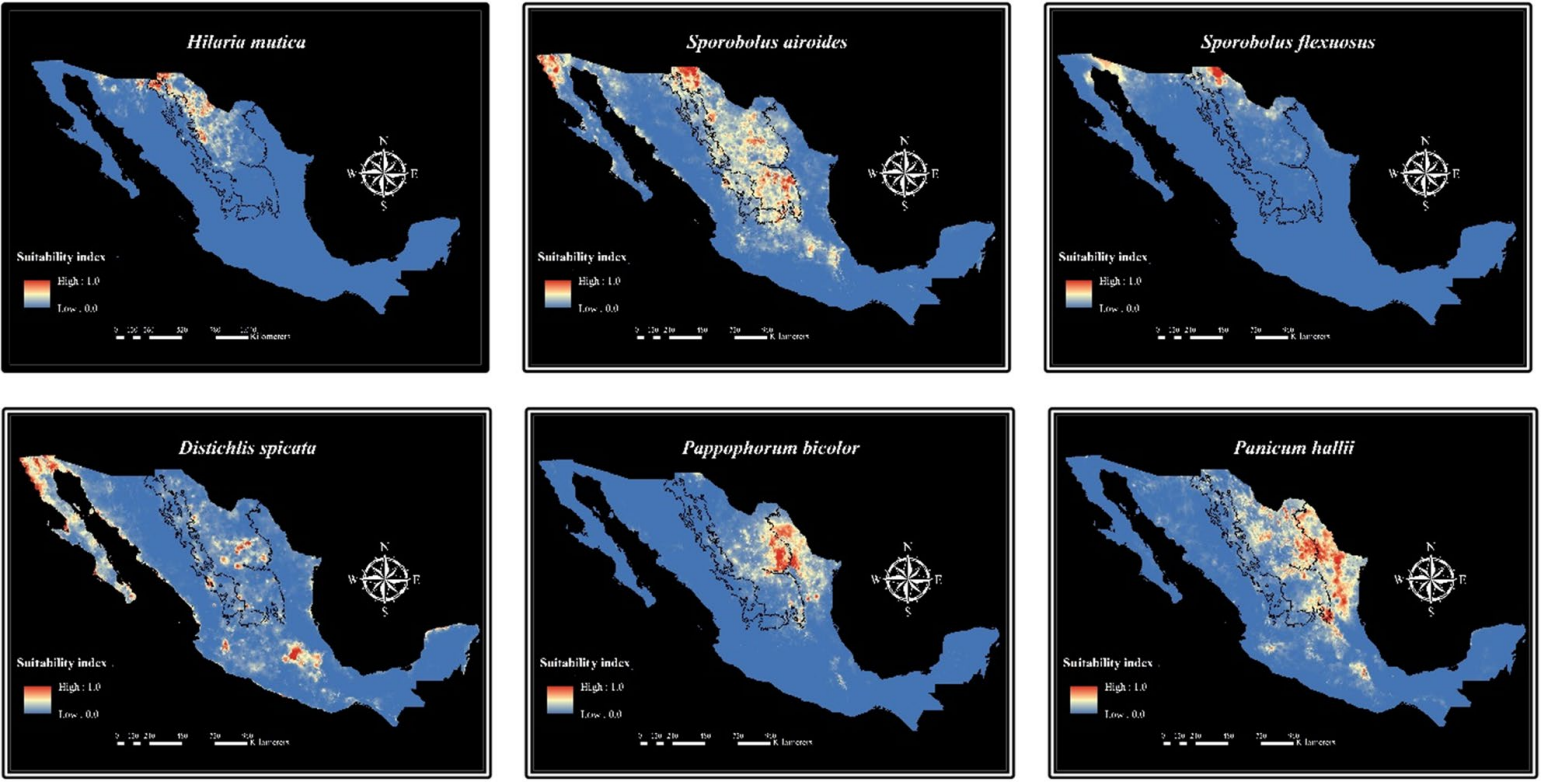
MaxEnt niche models for the potential distribution of six grass species that can be used for rangelands restoration in saline-sodic soils in Mexico. Relative environmental suitability for the species ranges from 0 (blue) to 1 (red). Black lines illustrate the grasslands (left) and shrublands (right) distribution ranges.

Species from both, groups 3 and 4, are adapted to high-pH soils. However, their resistance to soil sodicity varies. According to the response curves of the environmental niche models, *Hilaria mutica* and *Pappophorum bicolor* are vulnerable to sodicity but adapted to calcareous soils. Meanwhile, the rest of the species are highly resistant to sodicity (Fig. 4). Figure 5 shows the habitat suitability range of the species from Groups 3 and 4. The species from these groups had higher suitability in the shrublands than in the grasslands region.

## Discussion

Results from this study suggest that 27 from 49 species may have potential to be used for revegetation of degraded rangelands. Meanwhile, ten species may be adequate for revegetation of degraded grasslands, eleven for shrublands and six species for areas with sandy-alkaline soils.

These results demonstrated that the habitat suitability and environmental aptitude vary among the species evaluated, and thus, their potential use in rangelands restoration. The aforementioned is consistent with previous studies suggesting that environmental niche modeling of grass species can be a suitable method for biodiversity conservation and rehabilitation of rangeland ecosystems^13,28–30^. Such a method provides an estimation of the suitable areas and environments where the species can be planted. This information is of great importance for managing restoration efforts^14^.

*Bouteloua curtipendula*, *Bouteloua hirsuta*, *Bothriochloa barbinodis*, *Aristida divaricata*, *Bouteloua gracilis, Paspalum distichum, Setaria parviflora, Eragrostis intermedia*, *Disakisperma dubium*, *Bouteloua chondrosioides*, *Muhlenbergia phleoides,* and *Heteropogon contortu*s were the species from Group 1 (Fig. 1). These species are distributed in areas with high precipitation, low temperatures, and soils rich in fertility. Also, they showed the highest habitat suitability range (greater than 75%) and high suitability in the grasslands region. Therefore, their use for revegetation of degraded grasslands may be adequate. *Bouteloua curtipendula* and *B. gracilis* are two of the species most utilized in grasslands restoration in northern Mexico^31,32^. The rest of these species are not commonly used for restoration in Mexico, though they present important productivity and adaptability traits, which make them good candidates to be incorporated as grass species with potential for their use in rangelands restoration in northern Mexico. For instance, *Bouteloua hirsuta* is considered an important ranch grass since it has a very good forage quality and is a dominant species^33,34^. *Bothriochloa barbinodis* has a good establishment capacity but moderate forage quality^35^. *Aristida divaricata* has a fair to poor forage quality but good establishment capacity in poor soils^36^. *Disakisperma dubium* produces high forage quality and has been used as pioneer species to provide forage in the initial years after reseeding while other grasses are becoming established^37,38^. Likewise, *Eragrostis intermedia* is an important early spring forage grass for livestock. It has good forage quality and is highly preferred by cattle^39^. In addition, this species is highly resistant to grazing during drought periods^40^. *Bouteloua chondrosioides* is a species with good forage value, and highly palatable for livestock. This species is also resistant to fire^41^. *Muhlenbergia phleoides* is considered an important grass in northern Mexico since it is resistant to grazing and produces good forage quality^42^. Thus, the aforementioned species can be considered as grass species with potential to be included in future grasslands restoration programs to increase their probability of success.

*Heteropogon contortus* can be a candidate in the selection process of grass species with potential for restoration. Even though it produces forage of moderate quality, it is able to get established in disturbed and poor soils. Nevertheless, this species is highly dominant in plant communities and has become invasive in south Texas^43^. The presence of *H. contortus* can indeed have a significant impact on some wildlife populations. Accordingly, Bielfelt^44^ reported that an increase in the density of *H. contortus* positively correlated with the density of breeding pairs among three obligate grassland species: Cassin's sparrow (*Peucaea cassinii*), Eastern meadowlark (*Sturnella magna*), and grasshopper sparrow (*Ammodramus savannarum*). The density of Mourning doves (*Zenaida macroura*) also experienced an uptick with the rising density of *H. contortus*. However, the presence of scissor-tailed flycatchers (*Tyrannus forficatus*) decreased in response to the presence of *H. contortus*. Wied et al.^45^ highlighted that grassland birds appear to be caught in a delicate trade-off where *H. contortus* is abundant. On the one hand, they enjoy enhanced nesting conditions, yet, on the flip side, this habitat offers a narrower range of food resources. Therefore, this species should only be used to rehabilitate highly degraded areas. According to the ENM of this species, the variables with the highest contribution to the habitat suitability of this species were precipitation seasonality (Bio15; 20.8%) and precipitation of the coldest quarter (Bio19; 9.9%) (Table 3). The suitability index for this species is higher in areas with high precipitation seasonality and low precipitation of the coldest quarter. These results are consistent with those reported by Mata et al.^46^, who pointed out that the possible causes of the invasiveness of *H. contortus* may be an increase of milder winters and changes in precipitation patterns.

Predicted distribution maps showed that Group 2 has its highest suitability in the shrublands region of Northern Mexico (Fig. 3). Species from this group are adapted to low rainfall, high temperatures, and alkaline soils. Thus, they can be used to revegetate shrublands. The species from Group 2 showing the highest potential distribution range (greater than 75%) and suitability in the shrublands region were: *Aristida purpurea*, *Bouteloua ramosa*, *Digitaria californica*, *Enneapogon desvauxii*, *Muhlenbergia arenicola, Scleropogon brevifolius, Setaria leucopila, Setaria macrostachya*, and *Leptochloa crinita*.

*Aristida purpurea* is a perennial grass with monoculture tendencies, as well as poor forage quality and palatability relative to other native grass species^47,48^. However, it may be an alternative to revegetate degraded shrublands given its high establishment capacity in disturbed or overgrazed areas^49^. *Bouteloua ramosa* is a dominant perennial grass^50^. *Digitaria californica* is a grass species distributed from the southern United States to Argentina. This species is characterized for being resistant to drought, dominant in desert lands, highly palatable to livestock, and for having a high germination percentage^51–53^. *Setaria leucopila* is a grass also distributed from the southern United States to Argentina. This species has a good establishment capacity in disturbed areas^54^ and possess a good forage productivity^55^. *Enneapogon desvauxii* is a dominant grass^56^, which inhabits grasslands and shrublands from the southern United States to Argentina. *Muhlenbergia arenicola* is a low palatability forage grass^57^; however, it has a high resistance to fire^58^. *Scleropogon brevifolius* is a xeric species that inhabits arid and semi-arid regions of the southwestern United States and northern Mexico, where it can be a dominant species^59^. *Setaria macrostachya* is a species with good forage quality and tolerant to frequent defoliation^37,60,61^. Due to their forage characteristics and adaptability to harsh environments, it can be considered as a potential grass species to be included in future shrublands restoration programs.

Another species with high suitability in the shrublands region was *Leptochloa crinita* (Fig. 3). This species is distributed from the southern United States to Argentina. It produces good quality forage and is resistant to both, drought and grazing. In addition, it has a high germination percentage and high forage production rates, as well as good establishment capacity in disturbed areas^54,62–64^. For these reasons, *L. crinita* is considered one of the most important grasses in northwestern Argentina and it has been used to revegetate degraded rangelands of the region^62,65^. However, this species is not commonly used for restoration in Mexico. Thus, *L. crinita* has the potential to be selected as a grass species for future rangelands restoration efforts in Mexico.

*Pennisetum ciliare* (buffel grass) was included as a control species since it is the most utilized species for rangelands revegetation in the arid and semiarid regions of central and northern Mexico^18^. The use of this species has been recommended in shrublands where the native grasses currently used in restoration cannot be established^66^. However, *P. ciliare* is also considered an invasive exotic species in Mexico due to the associated impacts on the native biodiversity^67^. This particular species exhibits a monoculture growth pattern, which has ecological consequences. This monoculture growth habit simplifies the overall vegetation structure, leading to a reduction in biodiversity and a decrease in available habitat for numerous wildlife species. In addition, the presence of this species can disrupt natural fire regimes and interfere with essential nutrient cycling processes^45^. Therefore, it is necessary to identify native grasses that can be used as an alternative to *P. ciliare* for shrublands restoration. *Setaria leucopila, Setaria macrostachya, Digitaria californica, Enneapogon desvauxii, Leptochloa crinita*, and *Aristida purpurea* presented a similar habitat suitability range than *P. ciliare* in the shrublands region (Fig. 3). These suggests these species may be an alternative to *P. ciliare* amd could be used in the revegetation of degraded shrublands in northern Mexico.

According to the results of the cluster analysis and the ENMs, the species from groups 3 and 4 appear to be highly tolerant to soil salinity (Fig. 1). Species from Group 3 (*Hilaria mutica*, *Sporobolus airoides*, and *Sporobolus flexuosus*) are tolerant to sandy-alkaline soils while species from Group 4 (*Distichlis spicata*, *Pappophorum bicolor*, and *Panicum hallii*) are better adapted to clayey or silty alkaline soils. Thus, they can be selected as grass species with the potential to be used in future efforts of restoration of rangelands with alkaline soils. However, it is important to consider the type of alkalinity to which these species are adapted. According to the response curves of the ENMs, the maximum probability of habitat suitability for *Sporobolus airoides*, *Sporobolus flexuosus*, and *Distichlis spicata* occurs in soils with pH higher than 9 and sodium content higher than 10 mEq/l, whereas the maximum probability of habitat suitability for *Panicum hallii* is in areas with soil pH from 7.9 to 8.5 and sodium content of around 5 mEq/l (Fig. 4). Therefore, these species can be considered highly tolerant to soil sodicity. Meanwhile, *Hilaria mutica* and *Pappophorum bicolor* can be considered as adapted to calcareous soils but susceptible to sodicity. The maximum probability of habitat suitability for these species is also in soils with a pH higher than 9 and calcium content higher than 20 mEq/l (Fig. 4).

On the one hand, results from the ENMs revealed that elevation is an important factor for the environmental niche modeling of grass species (Table 1). It was the variable with the highest contribution for 28 of the 50 grass species evaluated. These results are in agreement with findings obtained in similar studies reporting elevation as one of the top contributing variables in grass species^68–70^. On the other hand, soil variables such as calcium and carbon content were the most important factors for the enviromental niche of three and one species, respectively. In addition, including soil data in the analyses allowed us to identify species highly adapted to sandy or clayey-alkaline soils (Groups 3 and 4). This is in accordance with previous studies, which stated that variables like elevation, soil pH, and soil texture could reveal patterns in suitability that cannot be inferred only from climate factors^13,69^. Accordingly, results from this study demonstrate the importance of including soil data in the environmental niche modeling of grass species.

Finally, it is important to note that, in this study the regularization multiplier was set as default. By doing so, the risk of overfitting may have increased and affected the robustness of MaxEnt models. This parameter helps mitigate overfitting by penalizing overly complex models, encouraging simpler and more generalized solutions^71,72^. Thus, the results of our models may be taken with the appropriate caution. We believe future research should explore the impact of different values of the regularization multiplier in environmental niche modeling, particularly in studies focused on identifying grass species with potential for rangelands restoration.

## Conclusion

This study provided insights about the environmental tolerances of different grass species distributed in the rangelands of northern Mexico. That served to identify grass species with the potential to be used in the restoration of degraded grasslands, shrublands, or sites with alkaline soils; particularly, species not currently used, or commonly used in the past, in the restoration of rangelands in Mexico.

Ecologists, conservation planners, researchers, and range managers could use the outcomes and the predicted distribution maps as base information to triage restoration efforts. This can help to increase the probability of success of future rangelands restoration programs. This is of great importance since restorations are often costly in terms of financial investments and labor.

### Supplementary Information

Supplementary Information 1.

Supplementary Information 2.

## Data Availability

All data generated or analyzed during this study are included in this published article [and its supplementary information files].
